# Awareness, Treatment, and Control of Hypertension in Primary Health Care and Secondary Referral Medical Outpatient Clinic Settings at Enugu, Southeast Nigeria

**DOI:** 10.1155/2016/5628453

**Published:** 2016-12-06

**Authors:** Chioli Chijioke, Raphael Anakwue, Teddy Okolo, Esther Ekwe, Chukwuemeka Eze, Charles Agunyenwa, Nnamdi Nwosu, Christopher Amah, Kenneth Nwadike, Udunma Chijioke

**Affiliations:** ^1^Department of Pharmacology & Therapeutics, University of Nigeria Teaching Hospital, Enugu, Nigeria; ^2^Department of Medicine, University of Nigeria Teaching Hospital, Enugu, Nigeria; ^3^Department of Family Medicine, University of Nigeria Teaching Hospital, Enugu, Nigeria; ^4^Department of Paediatrics Surgery, University of Nigeria Teaching Hospital, Enugu, Nigeria; ^5^Dietary Intervention Research Group, Chiolive International Medical Research Organisation and University of Nigeria Teaching Hospital, Enugu, Nigeria; ^6^Department of Laboratory Sciences, University of Nigeria, Enugu, Nigeria

## Abstract

Essential hypertension is the most common noncommunicable disease (NCD), affecting more than half the adult population in many countries and being the major NCD contributor to the double burden of disease in developing countries. We undertook a survey of the hypertension awareness, treatment, and control in primary and secondary referral health care clinics in Enugu, Nigeria, and compared these data with those obtained in local community surveys. The prevalence of hypertension in the primary care clinic (9.2%) was lower than in a previously reported community survey (42.2%), while, in the referral clinic, 70.3% of patients attending were hypertensive. Hypertension awareness rates were 91.9%, 29.4%, and 93.2% in these respective health care settings. Treatment and control rates (89.9% and 72.9%) were better in the secondary care clinic than in the primary care centre (87.7% and 46.0%). (Chi-square analysis confirmed statistically significant differences between these rates (*p* < 0.05).) These data may form a useful index of health care system effectiveness in Nigeria. Possible reasons for the differences observed and effective strategies to address the waxing pandemic of hypertension are discussed.

## 1. Introduction

Hypertension is at the forefront of a waxing pandemic of noncommunicable diseases, which has been ascribed to the human lifestyle changes in recent decades that accompany increasing urbanization and industrialization (“Westernization”) worldwide [1]. Systemic hypertension has been estimated to claim 92 million disability-adjusted life years per annum, 80% of this global burden being borne by developing countries [2].

Thus, from being a comparatively rare condition in many parts of the world until about 50 years ago, systemic arterial hypertension has become the leading cause of global morbidity and mortality [3]. The proportion of the world population with high blood pressure has increased up towards and even beyond half the adult population in some African countries, within a few decades [4–6]. The prevalence, awareness, treatment, and control rates in developing countries are approaching the rates in developed countries [6].

The prevalence of hypertension varies from as low as 5% in rural India to as high as 70% in Poland [7]. Much of the preeminence of hypertension in the global burden of disease stems from a lack of awareness, treatment, and control of the disease. Dong et al. [8] reported figures from rural China as low as 29.5%, 20.2%, and 0.9%, respectively. Hajjar and Kotchen [9] report, from the USA, that hypertension awareness remained static between 1990 and 2000 at about 69%, while treatment rates improved from 52.4% to 58.4% and control rates from 24.6% to 31.0%. In an earlier overview of 24 studies, Marques-Vidal and Tuomilehto [10] reported that, in men, the frequencies of awareness, antihypertensive drug treatment, and BP control among all hypertensive patients varied between 23% and 93%, 5% and 89%, and 5% and 87%, respectively. In women, the frequencies ranged between 28% and 97%, 6% and 97%, and 0% and 97%, respectively. Marques-Vidal and Tuomilehto [10] conclude that improvements in awareness, treatment, and control mean that the “rule of halves” no longer applies to industrialized countries. However the overall global position is a dismal “rule of thirds”: only about a third of people with hypertension are aware of their condition. Of these, only about a third are on treatment, and, less than a third on treatment are actually controlled and protected from cardiovascular complications [4].

There are comparatively few data on hypertension awareness, treatment, and control in Nigeria. Omuemu et al. [11] reported dismal rates of 18.5%, 14.3%, and 4.2%, respectively, from a rural community in Edo state. Kadiri et al. [12] found that awareness of blood pressure status was only about 8% in an urban population sample at Ibadan. In a series of 56 patients admitted with malignant hypertension, 75% were not aware of being hypertensive [13]. In a hospital-based study, Ukoh [14] reported that 20.2% of admissions were hypertensive. 52.5% of them were aware of their hypertension; their mortality was 22.1%. Ulasi et al. [15] found the prevalence of hypertension in a market population in Enugu, Nigeria, to be 42.2%, with only 29.4% aware. This prevalence was rather higher than the prevalence of 32.8% which they reported for semiurban and rural communities in Enugu state [16].

Apart from community studies to document local rates for prevalence, awareness, treatment, and control of hypertension, it is also important to study the effectiveness of local health care systems in improving dismal rates of awareness, treatment, and control. Comparatively few such studies have been conducted in this area and on the patient and healthcare provider barriers to improving hypertension awareness, treatment, and control [17]. With a view to studying these barriers in a developing country, we resolved to document and report on hypertension awareness, treatment, and control within our local health care setting and compare our findings with those from local community surveys [15, 16]. Such a comparison would provide a useful index of healthcare system effectiveness in tackling the waxing pandemic of hypertension and associated NCDs.

## 2. Objectives

This study aims to ascertain the proportions of hypertensive patients presenting to the primary health care (GOPD) and secondary referral outpatient clinics at UNTH who

- are not aware that they have high blood pressure,
- are aware of their hypertension but are not taking the prescribed treatment,
- are taking their prescribed treatment but their blood pressure is not controlled.

A subsidiary aim was to compare the clinic hypertension prevalence and awareness rates with those found in local community surveys.

## 3. Methods

These studies were undertaken in the General Outpatients Department (primary healthcare setting) and the Medical Outpatients Department (secondary/tertiary referral clinic) of the University of Nigeria Teaching Hospital during the period 2010–2012. During clinic sessions, records were kept by filling a* pro forma *questionnaire (see the Appendix) for each hypertensive patient (defined as a patient who either is taking antihypertensive medication or has blood pressure exceeding 140/90 mm Hg (or 130/80 for those with additional vascular risk) on at least two different occasions within the past month. The main focus was systolic pressure, bearing in mind that “systolic pressure is all that matters” [18] for those above 50 years of age, who bear most of the burden of hypertension. Blood pressure was taken using the left arm, sitting position (after checking for equality of the radial pulses), or as the higher pressure from the two arms (if radial pulses unequal). Patients were deemed to be not taking prescribed treatment if they were not taking the prescribed drugs at all or only taking partial treatment. Drug adherence was also cross-checked by pill counting and packet inspections in relation to the original prescription and its date. Deciding on whether blood pressure was controlled or not relied on the criteria above (based on at least two measurements within the previous month), that is, 140/90 mmHg or below signified control (130/80 mmHg or below for those with additional risk for vascular disease).

SPSS version 17 was used for chi-square analysis to assess the significance of differences between the clinics in the rates of hypertension awareness, treatment, and control.

## 4. Results

Out of 730 patients seen in the Medical Outpatients Clinic (MOP, secondary care referral clinic) 513 (70.3%) were hypertensive. Out of these, 478 patients (93.2%) were aware of their hypertension; that is, 35 patients (6.8%) were not aware that they had high blood pressure. Adherence was ascertained in 444 of these patients who brought their medicines to the clinic: 399 of these (89.9%) were fully adherent to prescribed treatment, while 45 (10.1%) were not. Out of 398 hypertensives adherent to their medication, only 290 (72.9%) had their blood pressure adequately controlled.

1552 patients were seen in the General Outpatients Department (GOPD, primary health care clinic) out of whom 143 (9.2%) were hypertensive. 62 of these patients were randomly selected for detailed assessment using the questionnaire. Of these patients, 57 (91.9%) were aware of their hypertension and 50 of these (87.7%) were taking their prescribed treatment, of whom only 23 (46.0%) had their blood pressure adequately controlled.

## 5. Discussion 

With “essential” hypertension at the forefront of a galloping pandemic of chronic diseases, an important index of health care effectiveness would be the comparative rates of hypertension awareness, treatment, and control in the health care system and in the community. Quite encouragingly, the hypertension awareness rates in the primary care and secondary health care settings (91.9% and 93.2%) were much higher than in the community survey (29.4%: [15]) (see Table 1). It is possible that the fact that patients in the primary and secondary care settings were already in the health care systems made them more aware of their burden of hypertension when compared with the community study. It is nevertheless surprising, especially at the secondary care referral clinic, that there are still some patients unaware of their hypertension status, in spite of being within the health care system. The lower awareness in the primary care setting (compared to the secondary care clinic) may reflect “dilution” of regular clinic attenders who are aware of their blood pressure status, by less regular and new attenders who are not aware of their hypertension.

The treatment and control rates in the medical outpatient clinics in this study were on the whole much better than a third. The expectation of the “rule of thirds” [4] would therefore need to be confirmed by further community studies.

Previous review had shown that the treatment rate of hypertension ranged from 5% in a rural Nigerian community to 91.2% in urban North African populations [19, 20]. The review also pointed out that despite varying rates of awareness and treatment, the control rates were uniformly low and never exceeded 45% [21]. The rates of hypertension treatment and control were not sought in the community studies by Ulasi et al. [15, 16]. The study by Omuemu et al. showed high treatment and control rates of 77.3% and 29.2%, respectively [11].

Data from the secondary care referral clinic suggest that the level of awareness and adherence to treatment for hypertension are relatively high. The 72.9% rate of control of blood pressure (and by implication, the prevention of long term complications) in the secondary care referral clinic in our study is encouraging and comparable to what is obtained in high income countries [22] but remains unsatisfactory. A recent study at this clinic in the University of Nigeria Teaching Hospital [23] shows that there is a need to improve patients' knowledge, perceptions, and practices, as regards treatment adherence and lifestyle modification. This would help to improve blood pressure control.

The relatively low prevalence of hypertension in the primary care level or GOPD (9.2%) may reflect the fact that a much broader range of patients is seen in the GOPD, while the difficulties in controlling blood pressure in essential hypertension lead to a large concentration of these patients in the secondary/tertiary care referral clinic.

The data from the primary health care setting suggest satisfactory awareness of hypertensive status. However the rates of adherence to treatment and control of blood pressure are poor. As treatment adherence and blood pressure control are better in the MOP than in the GOPD, this would suggest that more effort is needed in the primary care setting, including appropriate lifestyle counselling and the prescribing of multiple combination antihypertensive therapy to help improve blood pressure control. Those patients who remain poorly controlled in the GOPD setting should be referred to the MOP for intensified efforts to investigate and control their hypertension.

Retraining of healthcare providers to enable them keep to current guidelines in the management of hypertension would also improve outcomes in the primary care level. The findings in the primary care setting in our study are not new, as this is the case even in some high income countries where many barriers at the healthcare system, health care provider, and patient levels are known to impede the prevention and control of hypertension. These barriers include lack of access to care, costly medications, overburdened healthcare providers, lack of treatment guideline adherence, low patient health literacy, and adverse side effects [24].

Out-of-pocket expenditure that is particularly prevalent in many low-middle-income countries will deter hypertensive patients from obtaining prescribed drugs easily. Universal health coverage, where health insurance is the driving force for financing, will enable hypertensive patients to access care whenever and wherever they need it, ensuring availability of antihypertensive drugs at all times.

Although the 72.9% control rate achieved in the referral outpatient clinic is encouraging, it is still far from an ideal 100% control rate for hypertension. It is unlikely that modern antihypertensive medication would enable 100% blood pressure control, perhaps partly because the drugs target the phenotypic expression of hypertension without addressing the abnormal gene-environment interaction which begets the disease. A randomized controlled clinical trial of personalized food avoidance dietary counselling has accordingly begun at our centre and seeks to address lifestyle (i.e., dietary factors) in a more focused manner than hitherto, thus abating abnormal gene expression, and hence the disease phenotype. Addressing abnormal gene expression by appropriate lifestyle intervention, combined with appropriate drug treatment (to abate phenotype), is more likely to achieve full control blood pressure than relying on pharmacotherapy alone.

## 6. Conclusion

Treatment to target and control rate of hypertension in Nigeria has remained less than optimal. The plausible explanation is that although the efficacy and effectiveness of lifestyle modifications and antihypertensive pharmaceutical treatment for the prevention of hypertension and concomitant cardiovascular disease have been demonstrated in randomized controlled trials, this scientific knowledge has not been fully applied in populations living in low- and middle-income countries. The health system factors, healthcare providers together with the patient factors, play significant roles in this regard [21].

## Figures and Tables

**Table 1:** Comparison of rates of hypertension prevalence, awareness, treatment, and control in the local market community, primary health care, and secondary care referral clinics.

Setting	Prevalence% (number of subjects)	Awareness% (number of subjects)	Treatment% (number of subjects)	Control% (number of subjects)
Community [15]	42.2% (290)	29.4% (202)	NA	NA
Primary health care	9.2% (143)	91.9% (57)	87.7% (50)	46.0% (23)
2° care referral clinic	70.3% (513)	93.2% (478)	89.9% (399)	72.9% (290)

## Appendix

### A. Introduction to Questionnaire

This study aims to ascertain the proportions of hypertensive patients

(i.e. on antihypertensive drugs OR blood pressure exceeding 140/90)^*∗*^

presenting to doctors at Enugu who:

(a) are not aware that they have high blood pressure.
(b) are aware of their hypertension but are not taking the prescribed treatment^*∗∗*^
(c) are taking prescribed treatment but their blood pressure is not controlled (blood pressure exceeding 140/90)

^*∗*^i.e. blood pressure exceeding 140/90 mmHg (or 130/80 for those with diabetes, chronic kidney disease, or sickle cell disease) on at least two different occasions. The blood pressure to be recorded is either that measure in the left arm, sitting position, or the higher of the two arms.

  
^*∗∗*^i.e. not taking the prescribed drugs at all, or only taking partial treatment

Name and phone no. of doctor seeing patient: —
Location: —
Full day & date: —

### B. Questionnaire

*Profile of Patient*

Surname (of patient): —
Forename: —
Hospital folder no.: —
Date of birth: —
Gender: —
Full address: —
Telephone numbers: —
Email address: —
Occupation: —
Marital status:—

*Please Draw a Circle around the Correct Answer (Yes or No)*

Have you previously been told that you have high blood pressure? (Yes/No)
If yes, then when were you told that you have high BP?
Are you aware that you currently have high blood pressure? (Yes/No)
What treatment are you currently taking for your high BP?
(Record here the full drug prescription and dosages that the patient is supposed to be taking. Encircle “Yes” or “No” to indicate drugs actually being taken.)
—currently taking.? Yes/No
—currently taking.? Yes/No
—currently taking.? Yes/No
—currently taking.? Yes/No
—currently taking.? Yes/No
—currently taking.? Yes/No

*Data Extracted from Folder*

Full name of patient: — Folder no.: —
—currently taking.
—currently taking.
—currently taking.
—currently taking.
—currently taking.
—currently taking.

Record here the patient's blood pressure and dates (at least two readings on two different dates): —

Record here the patient's clinical presentation in terms of confirmed symptoms, signs, investigations, differential diagnosis, and confirmed diagnosis (if any): —
